# Effect of Intense Exercise on the Level of Bacteroidetes and Firmicutes Phyla in the Digestive System of Thoroughbred Racehorses

**DOI:** 10.3390/ani11020290

**Published:** 2021-01-24

**Authors:** Wanda Górniak, Paulina Cholewińska, Natalia Szeligowska, Magdalena Wołoszyńska, Maria Soroko, Katarzyna Czyż

**Affiliations:** 1 Department of Environment Hygiene and Animal Welfare, Wrocław University of Environmental and Life Sciences, Chełmońskiego 38C, 51-630 Wroclaw, Poland; 2 Institute of Animal Breeding, Wrocław University of Environmental and Life Sciences, Chełmońskiego 38C, 51-630 Wroclaw, Poland; paulina.cholewinska@upwr.edu.pl (P.C.); 119635@student.upwr.edu.pl (N.S.); maria.soroko@upwr.edu.pl (M.S.); katarzyna.czyz@upwr.edu.pl (K.C.); 3 Department of Genetics, Wrocław University of Environmental and Life Sciences, Kożuchowska 7, 51-631 Wroclaw, Poland; magdalena.woloszynska@upwr.edu.pl

**Keywords:** microbiome, intense exercise, Thoroughbred, racehorses

## Abstract

**Simple Summary:**

The microbiome of the digestive system of both animals and humans is affected by many factors including diet, environment, age, physical condition, and genetic factors, as well as the “host effect”. Physical effort, as a factor influencing the physiological state of an animal, can affect the microbial composition of the digestive system. The research so far is largely based on human and laboratory animals, but not enough research has been done on the effect of physical effort on horse digestive system. The study conducted suggests that effort has a significant impact on the studied bacterial phyla level and that there is also variation in the proportions of the studied phyla between individual horses, as well as “reacting” to changes in different ways, which also suggests the occurrence of a phenomenon called “host influence”.

**Abstract:**

Exercise significantly affects the body of both animals and humans, including the composition of the digestive microbiome. This study aimed to determine the changes in the composition of the most numerous bacterial phyla (Firmicutes and Bacteroidetes, as well as the level of the *Lactobacillaceae* family) in the digestive system of horses under the influence of physical effort. The study included a group of 17 Thoroughbred racehorses at the age of 3 years, fed the same forage, from whom feces samples were collected individually before and 48 h after physical effort. The obtained samples were subjected to DNA isolation and RT-PCR analysis. The results showed a significant increase in the level of both phyla after exercise compared to the state before physical effort; there were no such differences in the level of facultative aerobes, i.e., the *Lactobacillaceae* family (although a decreasing tendency was found after exercise). In addition, the analysis of the level of the studied phyla indicates individual differences in horses’ response to the effort.

## 1. Introduction

People and animals have their own unique set of microorganisms, which exhibit complex and multi-level interactions [1]. The equine intestinal microbiome consists of bacteria, protozoa, fungi, and archaea, which interact with each other and contribute to the proper functioning of the digestive and immune systems [1,2]. The main bacterial phyla (which are the most abundant in the digestive system compared to other microorganisms) include Firmicutes, Bacteroidetes, and Proteobacteria [3,4,5]. Horses are herbivores having a certain hindgut (caecum and colon) microbiota, enabling forage utilization for optimal nutrition, which provide a significant part of the daily energy requirement due to plant material fermentation [1]. The intestinal microflora in horses can be modified by diseases of the gastrointestinal tract [6], diet change [7,8], use of feed additives [9], or medications [10,11].

As a result of intense exercise, there are many reactions inside the body, including inter-compartmental fluid shifts, changes in autonomic tone, and increases in a sympathetic drive, which alters motility and decreases blood flow to the splanchnic region, which in turn allows an increase in blood flow to the skin, muscles, and other vital organs [12,13,14]. These temporary disturbances in blood flow lead to a lack of adequate oxygenation of the digestive system, including the intestinal mucosa, which also results in changes in the number and variety of microorganisms in the human digestive systems [15]. This, in turn, can alter fermentation and increase the risk of metabolic disorders in horses [16].

There are few reports of changes in the gut microflora in athlete horses or racehorses caused by exercise, compared to mice, rats, or humans [17,18,19,20,21,22,23]. However, it has been proven that physical exercise changes the composition of intestinal microbes, increases the number of butyrate-producing bacteria, and causes the changes in abundance of the Firmicutes, Bacteroidetes, Actinobacteria, and Proteobacteria groups in rodents, dogs, horses, and humans [24]. A study on eight Thoroughbred racehorses in active race training showed that the fecal microbiota is characterized by great diversity (in the range 1200–3000 operational taxonomic units per individual) and is dominated by Firmicutes and Bacteroidetes phyla [25]. Conditioning programs and intense exercise are also a stress factor for horses, which changes the structure of the bacterial community in the colon. As exercise intensity increases, the number of microorganisms responsible for digesting fiber decreases, and thus, fiber digestibility decreases [26]. Janabi et al. [27] proved that physical training of horses affects the gut microbiota, especially in the initial stage of training. The exercise training group showed significant changes in the levels of Bacteroidetes, Proteobacteria, Spirochaetes phyla, Clostridium, *Dysgonomonas* spp., and *Treponema* spp. The results of another experiment showed that very intense, sharp physical effort did not affect fecal microbiome of Standardbred racehorses and that 12 weeks of exercise training did not alter that response [14].

The aim of the study was to determine the level of bacterial phyla of Firmicutes and Bacteroidetes (including *Lactobacillaceae* family) in the digestive system of Thoroughbred racehorses affected by intense effort.

## 2. Materials and Methods

All horses that qualified for the study were subjected to standard procedures without any harm or discomfort and therefore the study did not require the consent of the Local Ethical Commission for Animal Experiments at the Institute of Immunology and Experimental Therapy of the Polish Academy of Sciences in Wroclaw, Poland (Act of 15 January 2015 on protection animals used for scientific or educational purposes). Consent from all horse owners was obtained prior to the investigations.

### 2.1. Animals

The study was conducted on a group of 17 Thoroughbred racehorses (7 mares and 10 stallions), 3 years old, with an average body weight of 457 ± 23 kg. The horses were clinically healthy and in good condition. All horses had a similar level of fitness and were trained daily for flat racing at Partynice Race Course (Poland) during the 2020 season. The animals were housed in individual box stalls bedded on straw, without access to pastures, in one stable. All stalls measured approximately 9 m^2^ and were cleaned six times a week in the morning. All horses were acclimatized to the facility and accustomed to the management, diet, training, and equipment for one year. The animals were dewormed and vaccinated per standard veterinary practice. All of the horses were fed a concentrate (oat and pelleted grain supplement of the following composition: micronized wheat, chaff from oat straw with increased nutrient content, wheatfeed, distillers’ grains, micronized soya beans, molasses, soya oil, calcium carbonate, vitamins and minerals, calcined magnesite, sodium chloride (Baileys Racehorse Cubes, Baileys Horse Feed, Braintree, Essex, UK—Table 1) at 2.5% of body weight daily and roughage (grass hay ad libitum). Water and mineral salt (Lisal, Kłodawa, Poland) were provided ad libitum. The diet was followed for 6 months prior to the collection of the first stool sample and continued until the end of the study. On the race day, horses were not deprived of feed or water.

### 2.2. Exercise Training and Exercise Test

During daily training, horses trotted a distance of 1 km and cantered at distances of up to 3000 m on a racing court. After the training, horses were untacked in the stable and cooled down on an automatic horse walker for approximately twenty minutes. The daily training time was the same for all horses. The exercise test was participation in the 1900 m race on the Partynice Race Course (Poland). The time to cover this distance was within 2′01.10–2′10.00. The last race or high speed training session prior to the race was two weeks earlier for all horses. The training and race were carried out within the same time frame.

### 2.3. Sample Collection

Fresh feces samples were collected individually from each animal prior to the race (no more than 24 h before starting the race) and 48 h after the race [26]. Each feces sample was collected into sterile containers (15 mL) immediately after defecation as described by Mach et al. [28]. Then, they were cooled down to the temperature of 4 °C for the time of transport (15 min) and then frozen at −26 °C for further analysis (1 week). All samples were collected at the same time of the day.

### 2.4. DNA Isolation

A Genomic Mini AX Stool kit (A&A Biotechnology, Gdańsk, Poland), modified by the addition of mutanolyzes and lysozyme, was used for DNA isolation. After an isolation, the quality of the obtained DNA was checked with a NanoDrop 2000 spectrophotometer from Thermo Scientific (Wilmington, NC, USA). The average DNA content was 140–150 µg/µL. The level of impurities in the samples for parameter 260/230 was 2.0–2.2, and for parameter 260/280 it was 1.8–2.0. In the case of high impurity levels or possibly low-quality DNA, the samples were re-isolated or cleaned with a Clean-up Concentrator (A&A Biotechnology).

### 2.5. Real-Time PCR Analysis

Real-time PCR analysis was performed with the use of a Bio-Rad CFX Connect 96 Touch apparatus with the help of a SsoAdvanced™ Universal SYBR^®^ Green Supermix kit (Bio-Rad Laboratories, Inc., Hercules, CA, USA) at a volume of 10 μL in 3 technical repetitions (Table 2). A no template control (NTC; without DNA sample, only primers) test was additionally performed for each gene. The real-time PCR analysis strategy was based on the amplification of primer specific for the tested phyla against the reference primer for all bacteria. The reference primers were 16S universal eubacterial genes 530F (5′-GTC CCA GCM GCN GCG G) and 1100R (5′-GGG TTN CGN TCG TTG) [29], for the Firmicutes phylum these were 16S 928F-Firm (5′-TGA AAC TYA AAG GAA TTG ACG) and 1040FirmR (5′-ACC ATG CAC CAC CTG TC), and for Bacteroidetes these were 16S 798cfbF (5′-CRA ACA GGA TTA GAT ACC CT) and cfb967R (5′-GGT AAG GGT TCC TCG CGT AT) [30]. For the *Lactobacillaceae* family the used primers were 16S, lac1 forward (5′-AGC AGT AGG GAA TCT TCC A), and Lac2Seq (5′-ATTTCACCGCTACACATG) [31].

A standard curve was prepared for the primers tested to determine the performance of individual genes. A sample dilution of 10^−6^ from the 10^−2^ to 10^−6^ series of dilutions was selected for analysis. The analysis was performed according to a protocol of 40 cycles: polymerase activation and DNA denaturation 95 °C (3 min), denaturation 95 °C (15 s), annealing 60.5 °C (15 s), extension and plate reading at 72 °C (40 s). The analysis of the melting curves for the samples was performed at temperatures ranging from 65 °C (5 s) to 95 °C (0.5 °C increments in 2 s). The data were then compiled using CFX Maestro software (Bio-Rad Laboratories, Inc., Hercules, CA, USA).

The sample with a DNA level of 150 μg/μL and impurities at a level in line with the above-mentioned standards was an arbitrary calibrator. CFX Maestro calculated the results from the number of reference gene matrixes and the differences at the relative normalized expression (ΔΔCq–RNE) phylum level, taking into account the amplification efficiency levels of individual genes. The performance of individual primers was correct (according to the standards established by Bio-Rad Laboratories) and amounted to 88.4% for Firmicutes, 101% for Bacteroidetes, and 98.7% for the *Lactobacillaceae* family, and in the case of Universal it was 92.4%.

### 2.6. Data Analysis

Statistical analysis was carried out with the use of Statistica software (v. 13.3, StatSoft Inc., Tulsa, OK, USA). The data distribution was verified using a Shapiro–Wilk test. As the distribution was normal (Bacteroidetes, *Lactobacillaceae*) the student’s *t*-test for dependent samples was used, and in the case of no normal distribution (Firmicutes), the Wilcoxon pair test (*p* > 0.05) was used.

## 3. Results

**Figure 1:**
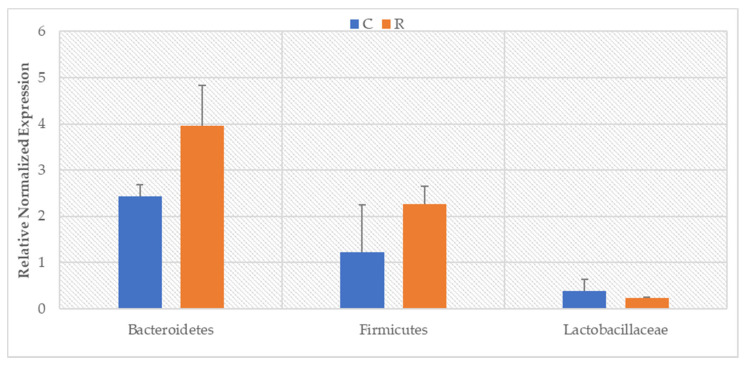
The level of bacteria (RNE) in the feces of animals before exercise (C) and after race (R).

The performed real-time PCR analysis showed significant differences in the level of the examined bacterial phyla. The tested animals were characterized by significantly lower levels of the Bacteroidetes and Firmicutes groups before intense exercise compared to the period after exercise (Figure 1: Bacteroidetes: *p* = 0.0177; Firmicutes: *p* = 0.0129). Although no significant changes in the level of the *Lactobacillaceae* family (Firmicutes phylum) were found, a downward trend was noted during the exercise period.

**Figure 2:**
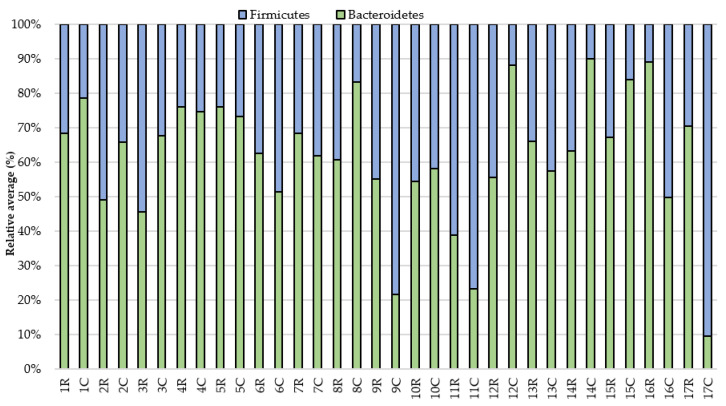
The relative average of the studied groups in the feces of animals before (C) and after race (R).

Then, the results for individual horses were analyzed. The pre-exercise analysis showed a greater proportion (over 60%) of the Bacteroidetes phylum in relation to Firmicutes in 10 horses compared to sampling after the exercise. The results also demonstrated that the share of Firmicutes increased and the share of Bacteroidetes decreased in 8th (horse No.) individuals after the race, while in the 6th the situation was the opposite. In the case of two individuals (No. 4, 5), the relationship between the phyla did not change (Figure 2). However, despite the similarities in the studied groups, differences in the level of the studied clusters can be noticed both at rest and after intense exercise.

Additionally, in seven individuals with a higher proportion of Firmicutes than Bacteroidetes before the exercise, the level of Firmicutes and an increase in Bacteroidetes decreased. The analysis of individual animals also showed individual differences, regardless of the effort. The differences in the level of the studied phyla in the digestive system between some individuals were significantly noticeable, such as between No. 1 and No. 17. Subject No. 1 was characterized by a higher level of the Bacteroidetes phylum than Firmicutes, while subject No. 17 showed the opposite phenomenon.

**Figure 3:**
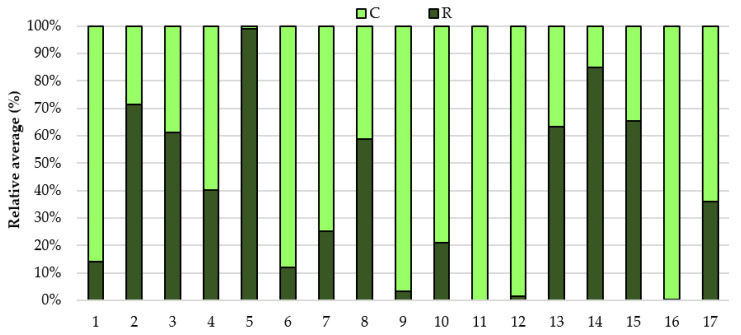
The relative average of the *Lactobacillaceae* family in the feces of the animals before (C) and after race (R).

The analysis of the *Lactobacillaceae* family also showed significant individual differences and changes in the family level depending on the effort. More than half of the horses were characterized by a higher share of the studied family in the digestive system before exercise than after. Additionally, it was observed, as in the case of the tested level of bacterial phyla, that regardless of the effort, individual differences were visible in the level of the studied family (Figure 3).

## 4. Discussion

Physical effort largely affects the physiological state of the body. Most studies of this type were carried out on humans and laboratory animals. Moderate exercise has a positive effect on the stimulation of the immune system, however, in the event of intense exercise, its function may be impaired, which may increase the risk of infection. In the case of people, there is often a phenomenon called the “open window effect” for pathogenic microorganisms, which lasts up to 72 h after the end of exercise [27,32]. It is generally believed that moderate (one-time or regular) exercise can stimulate the body’s immune system [32,33]. In addition, the studies conducted so far on horses suggest that the effects of moderate exercise may have immunomodulatory effects similar to those in humans [34,35,36].

Considering that the composition of the digestive system microbiome of both animals and humans is significantly influenced by the physiological state of the host, the effort and physiological changes associated with it may alter the structure of the microorganism community. In the study conducted, where exercise was performed by healthy individuals, additionally accustomed to this type of exercise, significant differences were shown in the level of Firmicutes and Bacteroidetes phyla; 48 h after exercise there was a significant increase in the level of both groups compared to the state before the exercise. The resulting changes in the level of the studied phyla may be related to the body’s response to exercise, i.e., changes in autonomic tone and increased sympathetic drive. At this time, there is also a decrease in blood flow in muscles, skin, and other organs, which in turn leads to a decrease (in the worst case, disappearance) of oxygenation of the intestinal mucosa, which could have a direct positive impact on the growth of bacteria in the digestive system of the tested horses [14,27,36]. It was shown in the study by Janabi et al. [14] conducted on Standardbred racehorses that an incremental exercise test increased the level of bacteria of the genus *Clostridium* and also showed changes in the level of the Bacteroidetes phylum, however, no changes in the level of Firmicutes were found. However, the study by Mika et al. [37] on rats showed changes in the levels of the Bacteroidetes and Firmicutes phyla—however, in this case there was a decrease in Firmicutes levels and an increase in Bacteroidetes levels, while a significant increase in both phyla abundance was found in our study. 

It should also be taken into account that the gastrointestinal microbiome acts as a specific immune system. As an immunologically active organ, it is constantly stimulated by both external (facultatively pathogenic microorganisms that enter with food) and internal epithelium (facultatively pathogenic microorganisms such as *E. coli*, *Salmonella*, or physiological changes in the body). Both their quantity and variety affect the proper condition of the intestinal mucosa and the development of intestinal villi. In turn, disturbances of the homeostasis of the digestive system microbiome may reduce the host’s immunity and thus increase the risk of inflammation and intestinal and parenteral infections [3]. Therefore, such a significant increase in the level of the studied phyla could be the result of the response of the digestive system microbiome to the factor that the body was exposed to, i.e., intense effort. The overall increase in the level of examined bacterial phyla may suggest that the physical effort stimulated the bacteria to proliferate, probably in order to increase the metabolism of nutrients, but further research on this issue is needed.

In addition, the obtained reactions of the animals’ organism to exercise could be related to previous occurrence of individual differences in the proportions of the studied phyla. The specific ecosystem of the digestive system, which includes the intestinal microflora, may be subject to modifications. This is due to environmental and genetic factors, such as age, race, diet, sex hormones, animal maintenance conditions, and geographical location [38,39,40]. Therefore, increasing research on the microbiome of the digestive system suggests that the individual is a significant factor, i.e., “host influence”. In human studies carried out as part of the Human Microbiome Project [5], the scientists suggest that each individual has its own microbiological composition of the digestive system, which is also visible in this study. As can be seen, despite the similarities in the level of the studied phyla, the individuals differed in their proportions.

However, due to the small amount of research carried out on horses, further analysis of the effects of both the individual and the effort on the gastrointestinal microbiome should be undertaken in the future, as the individual microbiological composition may also influence the horse’s response to physical effort.

## 5. Conclusions

In summary, exercise influenced the abundance of Firmicutes and Bacteroidetes groups, which may allow for a better adaptation of diet and maintenance conditions, as well as improve the intensity and quantity of training for racehorses in the future. Moreover, the results suggest differences in the horses’ individual responses to exercise, which may suggest a more individual approach to the animal in the future, in order to improve its health status and ability to adapt to exercise.

## Figures and Tables

**Table 1:** The nutrients in the pellet.

Ingredient	Content	Ingredient	Content
Digestible energy	13.50 MJ/kg	P	0.50%
Protein	13.00%	K	0.90%
Oil	5.50%	Mg	0.40%
Fibre	9.00%	S	0.30%
Ash	6.00%	Mn	75.00 mg/kg
Starch	26.00%	Fe	225.00 mg/kg
Sugar	4.00%	Cu (sulphate)	40 mg/kg
Ca	0.90%	Zn	130.00 mg/kg

**Table 2:** Proportion of PCR mix.

Component	Volume per 10 μL Reaction
SsoAdvanced™ Universal SYBR^®^ Green Supermix	5 μL
Forward and reverse primers	1 μL (0.8 μM)
DNA template	2 μL (0.04–0.015 × 10^−4^)
Nuclease-free water	2 μL

## Data Availability

The data presented in this study are available on request from the corresponding author. The data are not publicly available due to privacy.
